# Whooping-Cough, Treatment with Quinine

**Published:** 1874-04

**Authors:** 


Treatment of Whooping-Cough with Quinine.—Dr. B. F. Dawson has recently read a paper before the Medical Library and Journal Association of New York, on "The treatment of Whooping-Cough with Quinine," of which the following is a summary:

In regard to the administration of so disagreeable a remedy, he found that, though frequently there was some difficulty in getting the children to take it, yet it was exceptional for them to resist after the first two or three doses, and in only a very few did it cause vomiting. The direction to give the children a piece of an orange, or a little sugar five or six minutes after taking the quinine, had doubtless much to do with their seeming willingness to take the "bitter medicine." For his own part he accepts the fungus theory of Dr. Letzerich as the correct explanation of pertussis, and in consequence considers it an affection of the mucous membrane of the pharynx and larynx, and the whooping as simply reflex, and the fact that almost all remedies given for other than their local effects have either signally failed or but partially succeeded, he thinks strengthens this hypothesis.

In conclusion, the speaker felt convinced that if the following rules are carefully observed, few, if any, will be disappointed in their results:

1st. Give the quinine—sulphate or hydrochlorate—dissolved by acid in pure water only. For children under three years, from five to eight grains, and for older children and adults, from ten to twelve grains to the ounce.

2d. Give not less than a teaspoonful *every hour*, or, at the longest, every two hours during the day, and whenever cough comes on in the night.

3d. Give nothing afterwards for some minutes to destroy the taste or wash out the mouth.

4th. Continue giving it, notwithstanding the first doses may be vomited.

5th. Be sure that the quinine is pure and thoroughly dissolved.

In the foregoing paper the author wished to be understood as advocating the value of quinine in curing the "whooping" chiefly, the cough in some of the cases lasting for some time after the whooping ceased, and which required the usual treatment for bronchial catarrh.

